# Client Proteins and Small Molecule Inhibitors Display Distinct Binding Preferences for Constitutive and Stress-Induced HSP90 Isoforms and Their Conformationally Restricted Mutants

**DOI:** 10.1371/journal.pone.0141786

**Published:** 2015-10-30

**Authors:** Thomas L. Prince, Toshiki Kijima, Manabu Tatokoro, Sunmin Lee, Shinji Tsutsumi, Kendrick Yim, Candy Rivas, Sylvia Alarcon, Harvey Schwartz, Kofi Khamit-Kush, Bradley T. Scroggins, Kristin Beebe, Jane B. Trepel, Len Neckers

**Affiliations:** 1 Urologic Oncology Branch, Center for Cancer Research, National Cancer Institute, National Institutes of Health, Bethesda, Maryland, United States of America; 2 Developmental Therapeutics Branch, Center for Cancer Research, National Cancer Institute, National Institutes of Health, Bethesda, Maryland, United States of America; 3 Radiation Oncology Branch, Center for Cancer Research, National Cancer Institute, National Institutes of Health, Bethesda, Maryland, United States of America; University of Pittsburgh, UNITED STATES

## Abstract

The two cytosolic/nuclear isoforms of the molecular chaperone HSP90, stress-inducible HSP90α and constitutively expressed HSP90β, fold, assemble and maintain the three-dimensional structure of numerous client proteins. Because many HSP90 clients are important in cancer, several HSP90 inhibitors have been evaluated in the clinic. However, little is known concerning possible unique isoform or conformational preferences of either individual HSP90 clients or inhibitors. In this report, we compare the relative interaction strength of both HSP90α and HSP90β with the transcription factors HSF1 and HIF1α, the kinases ERBB2 and MET, the E3-ubiquitin ligases KEAP1 and RHOBTB2, and the HSP90 inhibitors geldanamycin and ganetespib. We observed unexpected differences in relative client and drug preferences for the two HSP90 isoforms, with HSP90α binding each client protein with greater apparent affinity compared to HSP90β, while HSP90β bound each inhibitor with greater relative interaction strength compared to HSP90α. Stable HSP90 interaction was associated with reduced client activity. Using a defined set of HSP90 conformational mutants, we found that some clients interact strongly with a single, ATP-stabilized HSP90 conformation, only transiently populated during the dynamic HSP90 chaperone cycle, while other clients interact equally with multiple HSP90 conformations. These data suggest different functional requirements among HSP90 clientele that, for some clients, are likely to be ATP-independent. Lastly, the two inhibitors examined, although sharing the same binding site, were differentially able to access distinct HSP90 conformational states.

## Introduction

**Fig 1:**
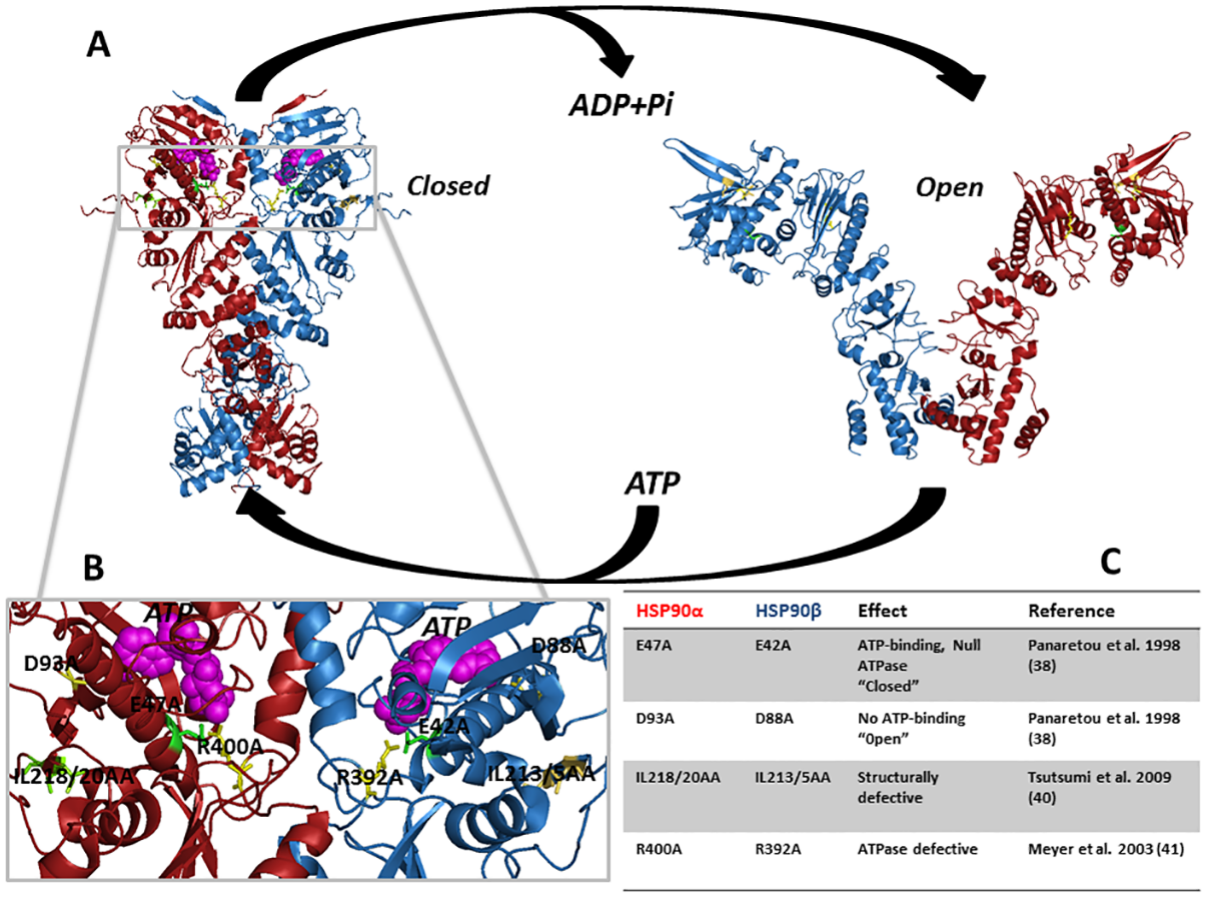
HSP90 structure and the chaperone cycle. **(A)** HSP90 ATPase-driven chaperone cycle: Depiction of the “closed” and “open” states of HSP90 fueled by ATP binding and hydrolysis. Image created in PyMol with PDB files 2IOQ and 2CG9. **(B)** The ATP-binding N-domain and relative location of conformational point mutants: Representative homologous location of human point mutants shown in yeast Hsp82 (PDB: 2CG9). Red backbone depicts HSP90α; blue backbone depicts HSP90β. **(C)** List of HSP90α and HSP90β conformational mutants and their functional descriptions.

The molecular chaperone heat shock protein 90 (HSP90) has been conserved throughout evolution, and functions primarily by coupling ATP hydrolysis to a cycle of structural rearrangements that drives the binding, folding and release of client proteins (Fig 1A) [1] [2]. Encoded by two different genes, HSP90α and HSP90β are the result of a gene duplication event that occurred early in the evolution of eukaryotes [3]. HSP90α is encoded by the *HSP90AA1* gene on human chromosome 14q and is induced in response to proteotoxic stress, inflammation and other cellular stimuli [4] [5]. HSP90β is encoded by the *HSP90AB1* gene on human chromosome 6p and is constitutively expressed. The two isoforms have evolved distinct functions despite sharing over 85% sequence identity [6–9] [10] [11]. Numerous drug discovery efforts have targeted this ATP-fueled molecular machine [12]. HSP90 inhibitors display preferential activity toward malignant or rapidly proliferating cells and have been found to concentrate and persist in tumor cells for an extended period, and these drugs have been extensively evaluated in the clinic [13] [14–16]. However, the drug binding pockets in HSP90α and HSP90β are very similar and pharmacologic approaches to specifically inhibit one isoform and not the other have yet to be successful [17].

HSP90 is predicted to interact with 7% of the transcription factors (TFs) in the human genome [18]. The stress activated TFs heat shock factor 1 (HSF1) and hypoxia inducible factor 1α (HIF1α) are HSP90 clients [19] [20]. HSF1 is a master regulator of stress-induced transcription and is often referred to as a guardian of the proteome. Unfortunately, HSF1 is also found to be over-expressed in a large number of cancers where it promotes a cancer-specific transcription program [21]. HSP90 binding to HSF1 is understood to inhibit its transcriptional activity but the underlying mechanism remains undefined [22] [23] [24] [20]. HIF1α is a master regulator of hypoxia-induced transcription and is responsible for promoting angiogenesis and metabolic reprogramming within oxygen-deprived tumor masses. HSP90 interacts with HIF1α to regulate interaction with its dimerization partner ARNT, a requirement for transcriptional activity [25,26].

HSP90 is predicted to interact with as much as 60% of the protein kinases in the human genome. However, the affinity with which HSP90 interacts with each client kinase varies [18]. This variation in interaction strength is related to the structural stability of the kinase domain, with which HSP90 physically associates [27] [28]. The tyrosine kinases ERBB2 and MET strongly interact with HSP90 and are well-established drivers of tumorigenesis and metastasis [29].

Work by Taipale et al predicts that HSP90 interacts with up to 30% of mammalian E3-ubiquitin ligases [18]. The HSP90 interactors KEAP1 and RHOBTB2/DBC2 act as tumor suppressors [30,31]. KEAP1 functions primarily to regulate stability of the master anti-oxidant response transcription factor NFE2L2 [32]. The function of RHOBTB2 is less established although it is understood to promote CCND2 degradation while also maintaining expression of CXCL14 on normal epithelial cells [33] [34].

Using these six proteins, drawn from three distinct functional classes of HSP90-dependent clients, we compared relative binding preferences for each HSP90 isoform as well as preference to interact with a set of conformationally trapped chaperone mutants.

Finally, we determined the interaction profiles of both HSP90 isoforms and their conformational mutants with geldanamycin and ganetespib [35]. Geldanamycin, an antibiotic derived from *Streptomyces hygroscopicus*, was the first identified HSP90 inhibitor [36] [37],while ganetespib is a synthetic HSP90 inhibitor currently in Phase 3 clinical trial in cancer patients [14].

Our findings in this study identify unexpected diversity in both isoform and conformational binding preferences among these individual HSP90 clients and inhibitors.

## Results

In addition to the wild-type (WT) proteins, we utilized a set of previously characterized point-mutants of Hsp90α and β, where equivalent mutations in yeast Hsp90 do not to support growth (Fig 1B and 1C). The “closed” N-domain dimerized conformational mutants E47A (HSP90α) and E42A (HSP90β) strongly bind ATP but lack ATPase activity [38]. In yeast, these mutants trap HSP90 in a conformation that tightly binds client proteins [39]. The “open” N-domain undimerized conformational mutants D93A (HSP90α) and D88A (HSP90β) do not bind ATP and are considered to interact only weakly with client proteins [38]. The β-sheet 8 mutants IL218/20AA (HSP90α) and IL213/5AA (HSP90β) lack the ability to form the proper intramolecular interactions upon ATP binding [40]. Each of these mutants is located within the N-terminal ATP-binding domain of HSP90. The last pair of mutants, R400A (HSP90α) and R392A (HSP90β), are located in the middle domain and are ATPase defective [41,42] [43] These HSP90 WT and mutant proteins were expressed in cells and examined for their ability to bind client proteins by immunoprecipitation-western blot analysis. This was complemented by LUMIER (LUMinescence-based Mammalian IntERactome) [44] analysis that allowed us to compare the relative interaction strength of each WT HSP90 isoform for individual client proteins. Effects of each HSP90 isoform on kinase and transcription factor activity were also examined. Finally, the relative interaction strengths of the two inhibitors with each isoform and their respective conformational mutants were profiled.

### HSP90 isoform interactions with transcription factors HSF1 and HIF1α

**Fig 2:**
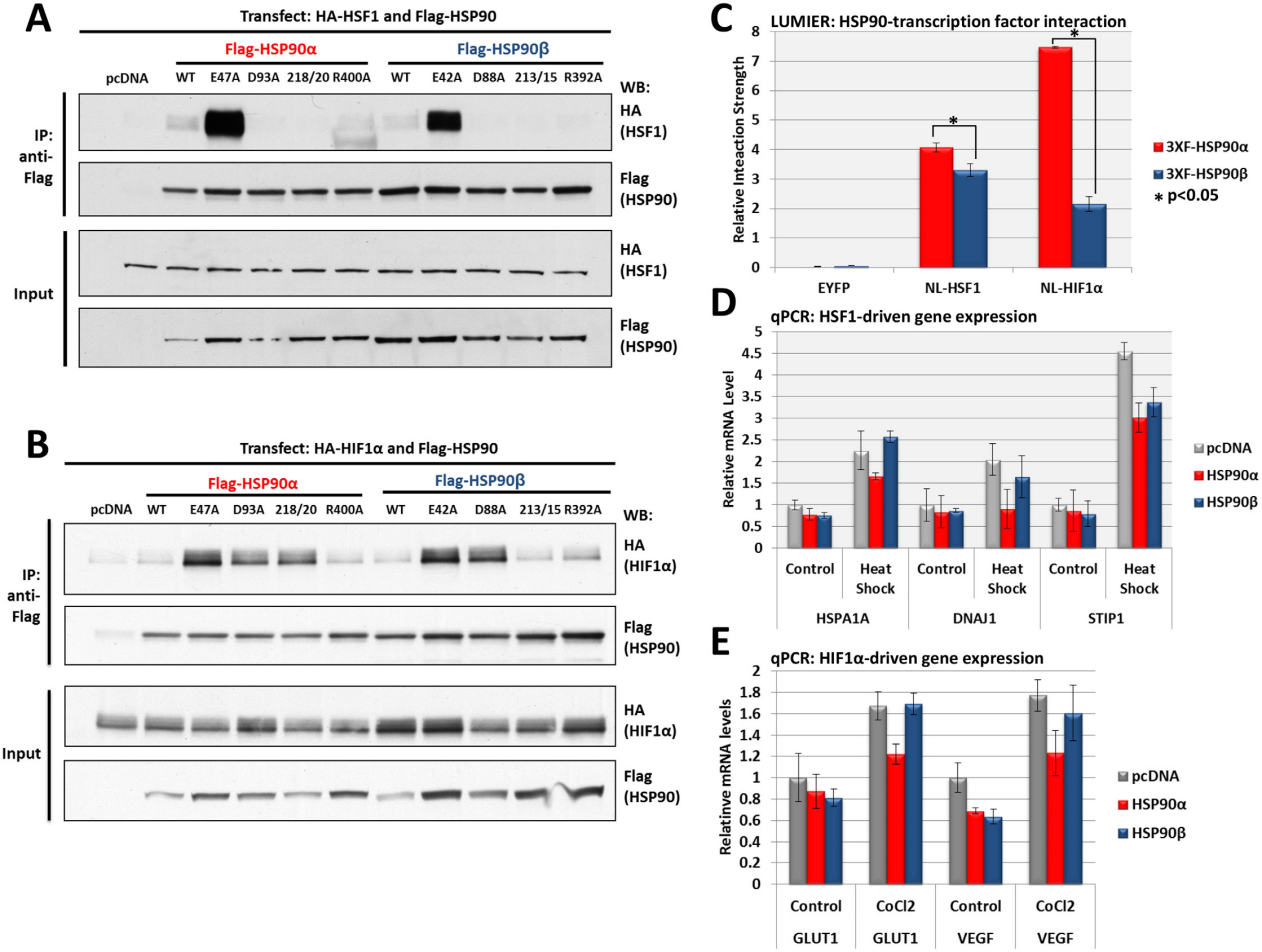
Interaction of HSF1 and HIF1α with HSP90 isoforms. **(A)** HSF1 interaction with HSP90 WT and mutants: HEK293 cells were transfected with HA-HSF1 and each FLAG-HSP90 construct, harvested, immunoprecipitated with anti-FLAG beads and western blotted for HSF1 interaction. Input lysates were normalized and run as controls. **(B)** HIF1α interaction with HSP90 WT and mutants: HEK293 cells were transfected with HA-HIF1α and each FLAG-HSP90 construct, harvested, immunoprecipitated with anti-FLAG beads and western blotted for HIF1α interaction. Input lysates were normalized and run as controls. **(D)** Measurement of the relative interaction strength of HSF1 and HIF1α with each HSP90 isoform by LUMIER: HEK293 cells transfected with HSF1 or HIF1α and each HSP90 isoform were harvested, applied to a 96-well anti-FLAG plate and assayed for luciferase activity. The difference in relative interaction strength of HSP90α and HSP90β for HIF1α (>3-fold) was statistically significant (p<0.05); the difference in relative interaction strength for HSF1, while much less, was also statistically significant (p<0.05) (see Methods). **(E)** Effect of each HSP90 isoform on heat shock induced gene expression: HEK293 transfected with each HSP90 isoform were heat shocked at 42°C for 30 minutes, allowed to recover for 2 hours, then harvested and assayed by qPCR for *HSPA1A*, *DNAJ1* and *STIP1* expression. **(F)** Effect of each HSP90 isoform on hypoxia-induced gene expression: HEK293 cells transfected with each HSP90 isoform were treated with 100 μM CoCl_2_ for 2 hours, harvested and assayed by qPCR for *SLC2A1* and *VEGFA* expression.

We transfected plasmids encoding FLAG-HSP90 WT and mutants along with HA-HSF1 or HA-HIF1α into HEK293 cells and allowed expression for 18 hours. Cells were then harvested and the interacting complexes were immunoprecipitated with anti-FLAG beads followed by western blot analysis. The “closed/ATP-bound” E47A and E42A mutants of both HSP90 isoforms bound most avidly to HSF1. In contrast, HSF1 binding to WT and other HSP90 mutants was quite weak (Fig 2A). These data indicate that while HSF1 clearly interacts with human HSP90, stable association is ATP-dependent and is thus restricted to a transient conformational state that, in humans, is not highly populated as shown by small-angle X-ray scattering and kinetic analysis [45] [46].

We observed a different HSP90 interaction pattern for HIF1α compared to HSF1 (Fig 2B). While HIF1α bound strongly to the “closed/ATP-bound” E47A and E42A mutants, it also bound to the “open” D93A and D88A mutants. HSP90α IL218/20AA also bound HIF1α while HSP90β IL213/5AA did not. All other HSP90 proteins, including both WT HSP90 isoforms, bound HIF1α poorly. These data suggest that HSP90 is capable of binding certain clients with equal avidity while occupying a variety of conformational states.

We used LUMIER analysis to compare the relative interaction strengths of HSF1 and HIF1α with each WT HSP90 isoform. We observed that HSP90α bound HIF1α with greater relative interaction strength compared to HSF1, while HSP90β bound HSF1 with greater relative interaction strength compared to HIF1α (Fig 2C). This was interesting, since HSF1 bound strongly only to the “closed/ATP-bound” conformational state of both HSP90 isoforms while HIF1α bound both the “closed/ATP-bound” and “open” mutants, suggesting that binding to more than one conformational state may increase overall apparent affinity for certain clients.

We also found that HSP90α bound both TFs with greater relative interaction strength than did HSP90β. The difference in relative interaction strength between HSP90 isoforms for HSF1 and HIF1α may lead to differential effects on TF activity. To test this possibility, we analyzed the stress-induced transcription activity of both HSF1 and HIF1α after co-expressing either HSP90 isoform. To examine HSF1 activity, cells were heat shocked for 30 minutes at 42°C, allowed to recover for 2 hours, harvested and analyzed for HSF1-driven gene transcription by quantitative PCR (qPCR). Heat shocked samples that overexpressed HSP90α consistently demonstrated reduced expression of the HSF1-driven genes *HSPA1A*, *DNAJ1* and *STIP1* compared to the pcDNA control. HSP90α repressed the expression of *HSPA1A* and *DNAJ1* to a greater degree than did HSP90β, while both isoforms repressed the expression of *STIP1* (Fig 2D).

To compare the effects of HSP90α and HSP90β on HIF1α-driven transcription we transfected plasmids for each isoform together with HIF1α into HEK293 cells and allowed them to express for 18 hours. At that time, 100 μM CoCl_2_ was added to induce pseudo-hypoxia. The cells were harvested 2 hours later and analyzed for HIF1α-driven gene transcription by qPCR. Pseudo-hypoxic samples that overexpressed HSP90α showed greater reduction in transcription of the HIF1α target genes *SLC2A1* and *VEGFA* compared to samples that overexpressed HSP90β (Fig 2E). Our findings are consistent with a model in which HSP90α, unlike HSP90β, may function in a negative feedback loop to regulate the activity of these stress-induced, HSP90-dependent TFs.

### HSP90 isoform interactions with tyrosine kinases ERBB2 and MET

**Fig 3:**
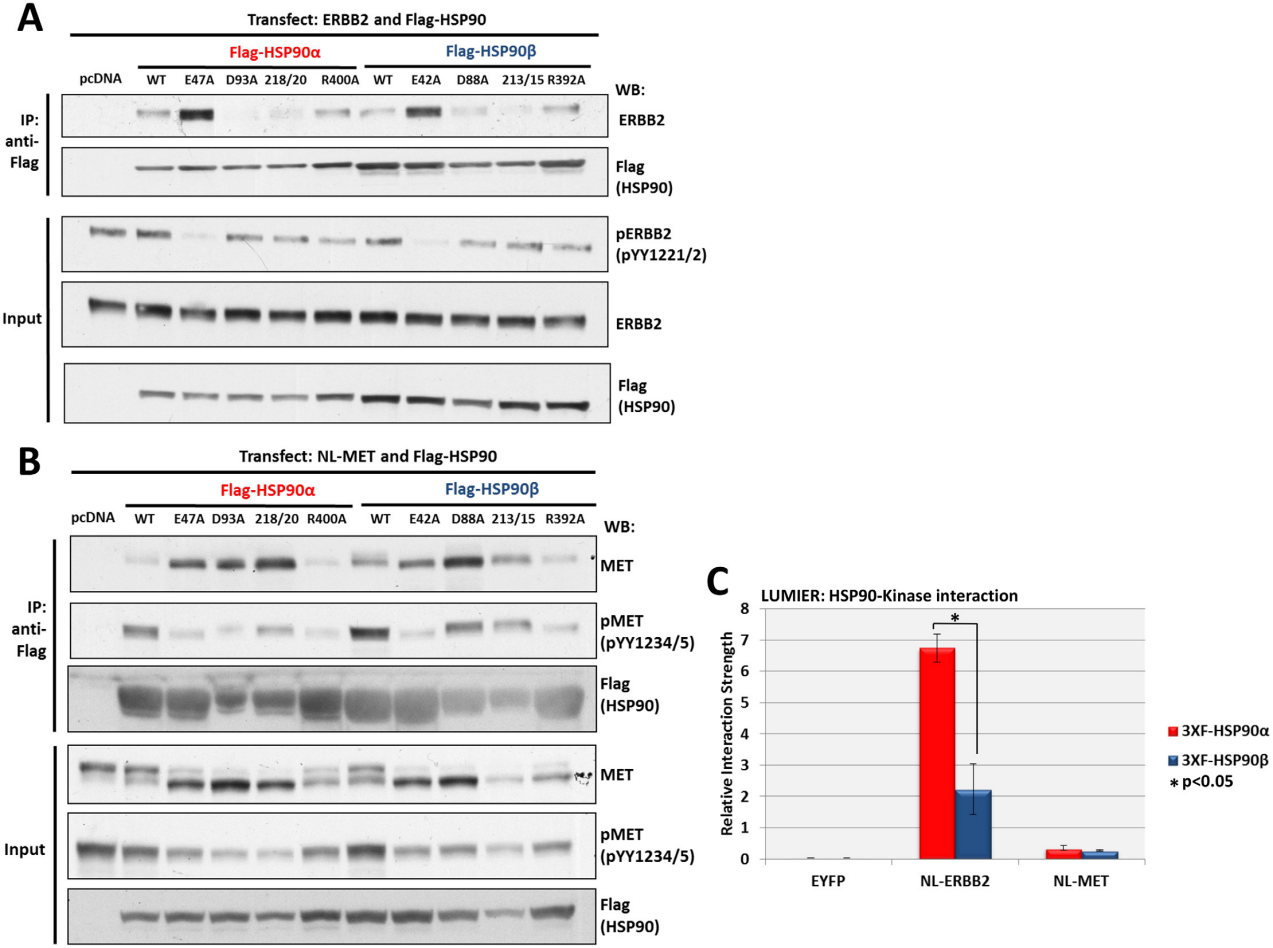
Interaction of ERBB2 and MET with HSP90 isoforms. **(A)** ERBB2 interaction with HSP90 WT and mutants: HEK293 cells transfected with ERBB2 and each FLAG-HSP90 construct were harvested, immunoprecipitated with anti-FLAG beads and western blotted for ERBB2 interaction and pY1221/pY1222 intensity. Input lysates were normalized and run as controls. **(B)** MET interaction with HSP90 WT and mutants: HEK293 cells transfected with MET and each FLAG-HSP90 construct were harvested, immunoprecipitated with anti-FLAG beads and western blotted for MET interaction and pY1234/1235 intensity. Input lysates were normalized and run as controls. **(C)** Measurement of the relative interaction strength of ERBB2 and MET with each HSP90 isoform by LUMIER: HEK293 cells transfected with MET or ERBB2 and each HSP90 isoform were harvested, applied to a 96-well anti-FLAG plate and assayed for luciferase activity. The difference in relative interaction strength of HSP90α and HSP90β for ERBB2 (>3-fold) was statistically significant (p<0.05) (see Methods).

Similar to the methods used to study these TFs, we co-transfected plasmids encoding FLAG-tagged WT and mutant HSP90 together with ERBB2 or MET tyrosine kinases in HEK293 cells. After 18 hours, cells were harvested and the interacting complexes were isolated by immunoprecipitation with anti-FLAG beads and analyzed by western blot. As was the case for HSF1, the HSP90 “closed/ATP-bound” E47A and E42A mutants interacted most strongly with ERBB2 (Fig 3A). ERBB2 also bound both WT HSP90 constructs at detectable levels along with the ATPase defective R400A and R392A mutants. To determine the effects of the HSP90 mutants on ERBB2 kinase activity we blotted for pY1221/1222, an ERBB2 auto-phosphorylation site. We observed that ERBB2 was auto-phosphorylated in all lysate inputs except for those that were transfected with the tightly binding E47A and E42A mutants. These data indicate that ERBB2 interaction with HSP90 is specific to a defined and transient state within the HSP90 ATPase cycle. Moreover, HSP90 binding represses ERBB2 kinase activity. This is consistent with earlier reports demonstrating rapid but transient increases in kinase activity, including that of ERBB2 [47] [48], following HSP90 inhibition [49].

Both WT HSP90 isoforms bound MET to some extent (Fig 3B). MET was most strongly bound by the “closed” E47A and E42A mutants, the “open” D93A and D88A mutants and the IL218/20AA and IL213/5AA mutants. The R400A and R392A mutants bound MET less robustly. To determine the effects of the HSP90 mutants on bound MET kinase activity we blotted for pY1234/1235 in the pull-down samples. Here we observed that MET was clearly auto-phosphorylated in both WT HSP90 samples and in cells transfected with HSP90β D88A, HSP90α IL218/20AA and HSP90β IL213/5AA. In all other samples, MET activity was greatly reduced. We also blotted for pY1234/1235 in the input samples and observed that MET was phosphorylated to some degree in every lane but most heavily in the pcDNA controls and samples transfected with both WT HSP90 isoforms. This was also reflected in the mobility shift of MET in the input blots. The data indicate that MET associates with multiple HSP90 conformational states, in contrast to ERBB2. As was the case for ERBB2, MET interaction with HSP90 suppresses its kinase activity.

Using LUMIER to compare the relative interaction strengths of ERBB2 and MET with each WT HSP90 isoform (Fig 3C), we observed that both HSP90α and HSP90β bound ERBB2 with greater affinity compared to MET. However, this difference may be related to the unexpected finding that MET overexpression increased the overall expression of both HSP90 isoforms, thereby reducing the apparent relative interaction strength (see methods for calculation of relative interaction strength). This was also reflected in the immonoprecipitation-western blot band intensities for HSP90 in samples co-transfected with MET. Similar to the TFs, ERBB2 bound HSP90α with greater relative interaction strength compared to HSP90β.

### HSP90 isoform interactions with E3 ubiquitin ligases KEAP1 and RHOBTB2

**Fig 4:**
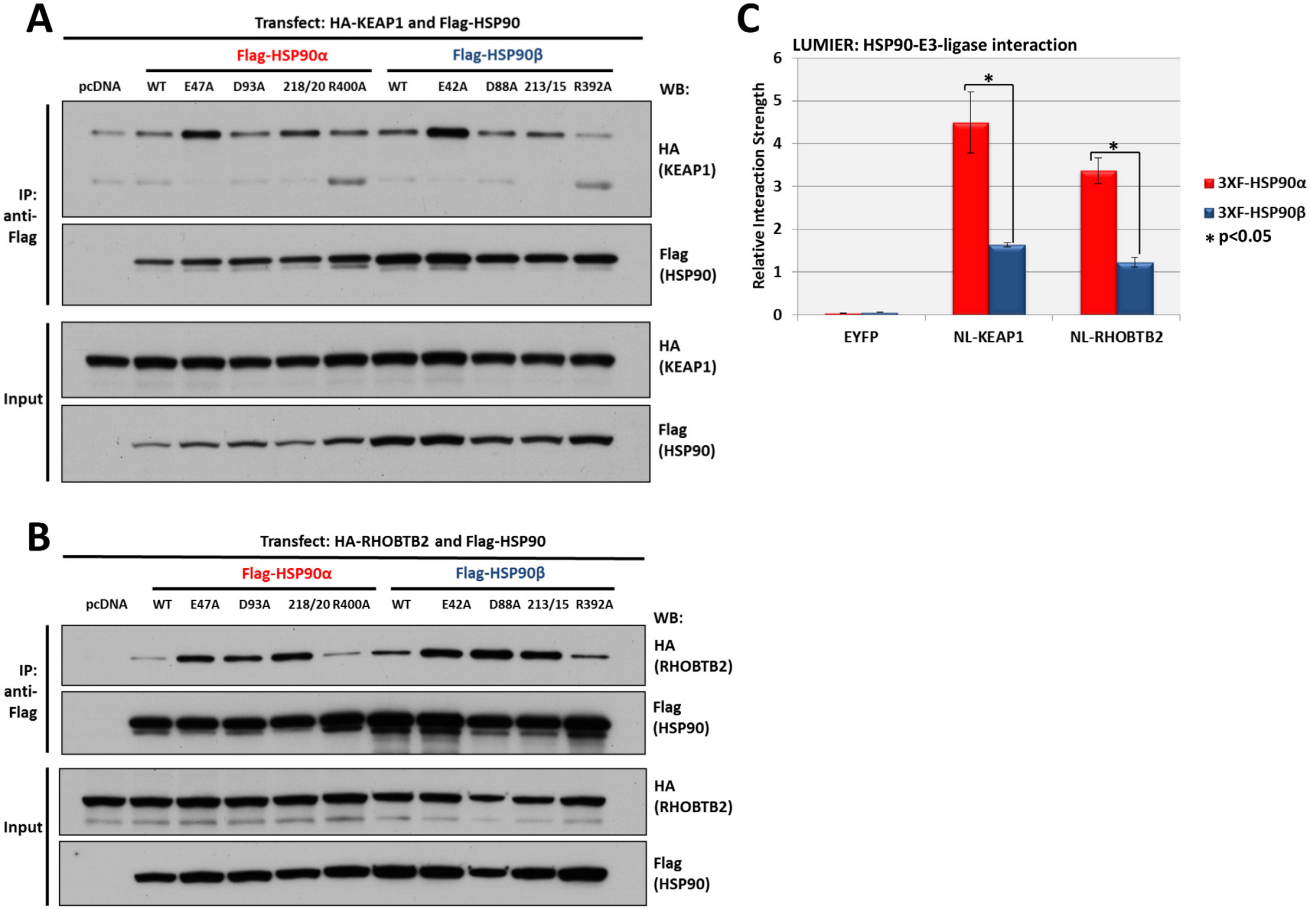
Interaction of KEAP1 and RHOBTB2 with HSP90 isoforms. **(A)** KEAP1interaction with HSP90 WT and mutants: HEK293 cells transfected with HA-KEAP1 and each FLAG-HSP90 construct were harvested, immunoprecipitated with anti-FLAG beads and western blotted for HA. Input lysates were normalized and run as controls. **(B)** RHOBTB2 interaction with HSP90 WT and mutants: HEK293 cells transfected with HA-RHOBTB2 and each FLAG-HSP90 construct were harvested, immunoprecipitated with anti-FLAG beads and western blotted for HA. Input lysates were normalized and run as controls. **(C)** Measurement of the relative interaction strength of KEAP1 and RHOBTB2 with each HSP90 isoform by LUMIER: HEK293 cells transfected with KEAP1 or RHOBTB2 and each HSP90 isoform were harvested, applied to a 96-well anti-FLAG plate and assayed for luciferase activity. The difference in relative interaction strength of HSP90α and HSP90β for KEAP1 and RHOBTB2 (each approximately 3-fold) was statistically significant (p<0.05) (see Methods).

We compared the HSP90 binding preferences of KEAP1 and RHOBTB2 using the methods described above. Each HSP90 construct bound KEAP1 above pcDNA background, although both E47A and E42A mutants associated most strongly (Fig 4A). Every HSP90 construct bound to RHOBTB2 above pcDNA background (Fig 4B). Moreover, most mutants bound RHOBTB2 more effectively than did WT HSP90, while R400A and R392A bound RHOBTB2 at a level similar to the corresponding WT isoform.

We used LUMIER to determine the relative interaction strength of KEAP1 and RHOBTB2 with each WT HSP90 isoform (Fig 3C). Similar to the previous two client sets, both KEAP1 and RHOBTB2 bound HSP90α with greater relative interaction strength compared to HSP90β. Moreover, KEAP1 bound both HSP90 isoforms with greater relative interaction strength than did RHOBTB2.

### HSP90 isoform interactions with geldanamycin and ganetespib

**Fig 5:**
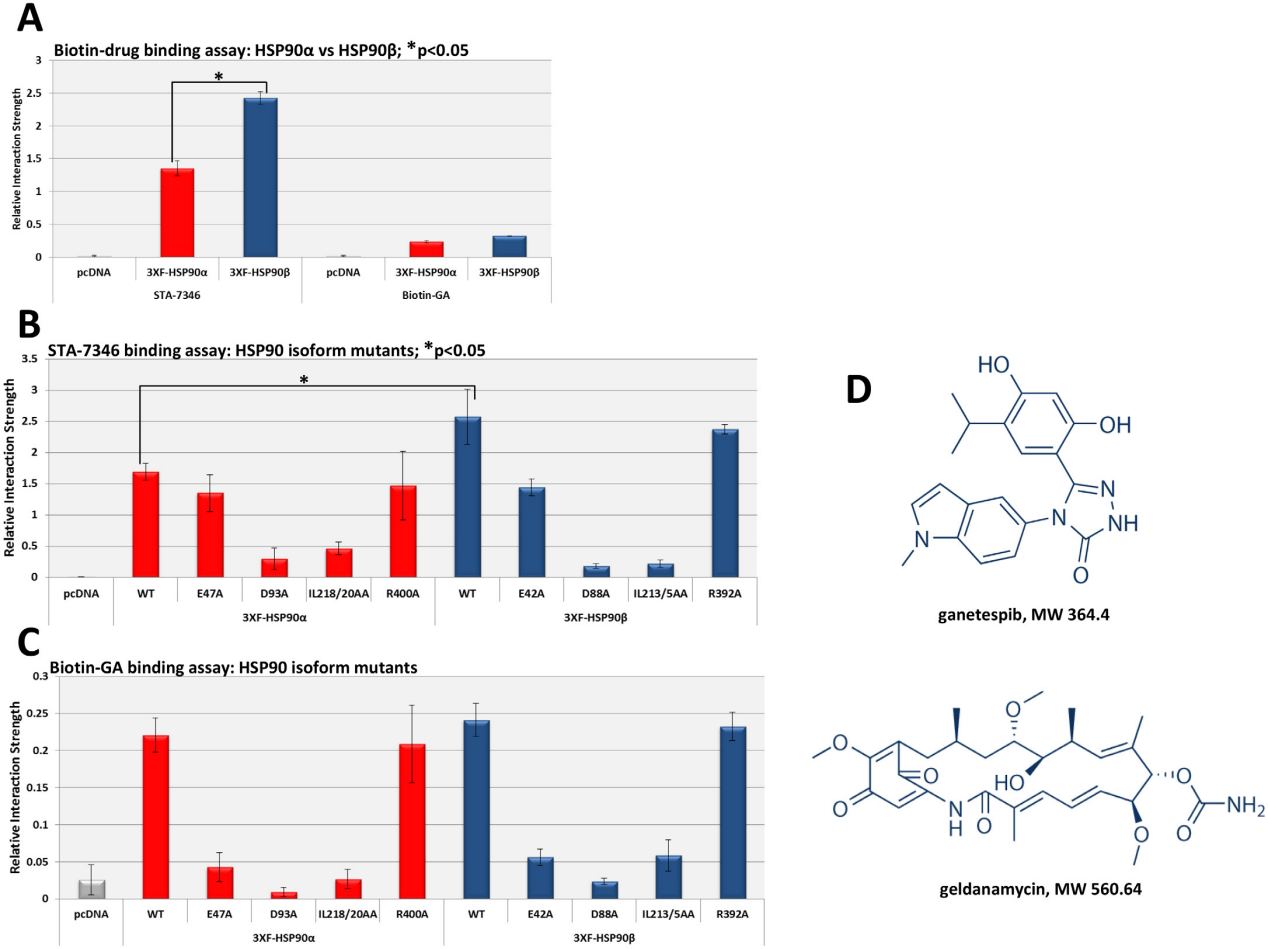
Interaction of HSP90 inhibitors with HSP90 isoforms. **(A)** Comparison of the relative interaction strength of STA-7346 and biotin-GA for each HSP90 isoform: HEK293 cells transfected with each HSP90 isoform were harvested, applied to an STA-7346 or biotin-GA coated streptavidin plate and assayed for interaction. The difference in relative interaction strength of HSP90α and HSP90β for STA7346 was statistically significant (p<0.05) (see Methods). **(B)** Relative interaction strength of STA-7346 with each HSP90 WT and mutant isoform: HEK293 cells transfected with each HSP90 construct were harvested, applied to an STA-7346 pre-bound streptavidin plate, and assayed for interaction. The difference in relative interaction strength of WT HSP90α and HSP90β for STA7346 was statistically significant (p<0.05), and comparable to the difference shown in panel A. **(C)** Relative interaction strength of biotin-GA with HSP90 WT and mutants: HEK293 cells transfected with each HSP90 construct were harvested, applied to a biotin-GA pre-bound streptavidin plate, and assayed for interaction (see Methods). **(D)** Chemical structures of ganetespib and geldanamycin: Images taken from selleckchem.com website.

Lastly, we compared the relative interaction strength of each HSP90 isoform for two inhibitors, geldanamycin and ganetespib. These ATP-competitive small molecule inhibitors share the same N-domain binding site and functionally prevent nucleotide-dependent N-domain dimerization and ATP-dependent chaperone activity [50]. We quantified binding of each HSP90 isoform to either biotinylated-geldanamycin (biotin-GA) or biotinylated-ganetespib (STA-7346). In contrast to the client proteins, HSP90β bound each inhibitor with greater relative interaction strength than did HSP90α (Fig 5A). Both HSP90 isoforms bound STA7346 with markedly greater relative interaction strength compared to biotin-GA, consistent with the fact that ganetespib has a much lower Kd and IC_50_ compared to geldanamycin [14]. Examination of the HSP90 N-domain mutants provided unexpected results (Fig 5B and 5C). The “closed” mutants E47A and E42A bound both drugs with less relative interaction strength than did their respective WT counterparts, but STA-7346 binding was much less compromised compared to that of biotin-GA. In fact, given the overall difference in relative interaction strength between the two inhibitors, STA-7346 bound significantly better to the “closed” E47A and E42A mutants than did biotin-GA to WT HSP90. Consistent with previous reports, the non-ATP binding “open” mutants D93A and D88A displayed the least drug binding activity [51]. Additionally, both of the “structurally defective” N-domain mutants, IL218/20AA and IL213/5AA, bound STA-7346 and biotin-GA with low relative interaction strength. This was unexpected because β-sheet 8 does not directly contact the ATP-binding pocket, suggesting long-range conformation effects on drug binding. In contrast, the ATPase defective middle domain mutants R400A and R392A bound both drugs with slightly reduced relative interaction strength compared to WT constructs. These data indicate that each HSP90 isoform may be divergently sensitive to N-domain inhibitors, and that the less bulky synthetic inhibitor ganetespib (MW 364.4) may be able to access HSP90 conformational states not available to the natural product geldanamycin (MW 560.64) (Fig 5D).

## Discussion

Taken together, these data suggest that certain unappreciated dynamics govern client and drug HSP90 interaction preferences in a cellular context. HSP90α and HSP90β differ in relative preference for both clients and two N-domain inhibitors. HSP90α bound clients across three distinct functional classes with greater relative interaction strength compared to HSP90β, while HSP90β bound inhibitors more strongly than did HSP90α. Remarkably, the less bulky synthetically derived inhibitor ganetespib was able to access HSP90 conformational states (e.g., the “closed” E47A and E42A states) previously considered inaccessible based on binding studies with natural product inhibitors. This ability, as well as superior binding affinity, may underlie the increased cellular potency of ganetespib and other 2^nd^ generation synthetic inhibitors. However, given the preference of these drugs for HSP90β over HSP90α, our data suggest that development of isoform-specific HSP90 inhibitors remains of interest.

Unexpectedly, we observed differences in client interaction preferences with a panel of conformationally restricted HSP90 mutants, even when the client proteins evaluated have similar biological functions. Conversely, we identified clients of different functional classes that share the ability to interact with similar HSP90 conformational states. For example, robust HSP90 interaction with HSF1 and ERBB2 appears to be ATP-dependent and likely occurs transiently under normal chaperone cycling conditions, while HSP90 interaction with HIF1α, MET, and the two E3-ubiquitin ligases is not as conformationally restricted. Although the biological significance of these findings is at present unclear, our data suggest that for some clients HSP90 may possess passive binding activity that is ATP-independent. Whether this reflects a previously proposed “holdase” or anti-aggregation function of HSP90 that may be inhibitor insensitive requires further investigation [52] [53] [54]. The ability of certain clients to interact with multiple HSP90 conformations, as well as a greater dependence of inherently unstable clients on HSP90 association (e.g., v-Src) may also explain the ease or difficulty in detecting dynamic HSP90/client interactions in cells [18] [10].

Additionally, our data confirm that the biological function of some HSP90 clients may be suppressed by strong interaction with conformationally restricted HSP90 mutants or distinct HSP90 isoforms [47,48,55] [49]. This was the case for ERBB2 and MET kinases and for the TFs HSF1 and HIF1α. For HSF1, these observations support a feedback model where HSF1-driven transcription of HSP90α may negatively regulate HSF1 activity. Our data are consistent with the hypothesis that HSP90 may function more to attenuate HSF1 transcription activity than to suppress HSF1 activation under non-stress conditions [20]. Although this needs to be confirmed in other cell types, our findings suggest that a re-examination of the role of HSP90 in modulating HSF1 activity, and the effect of inhibitors on this modulation, is necessary. In conclusion, the results of this study emphasize that detailed analysis of the conformational dependence of HSP90 interaction with its diverse clientele, and with inhibitors currently being evaluated in the clinic, is necessary to understand how best to target this molecular chaperone in cancer and other diseases.

## Methods and Materials

### Plasmids

pcDNA3-FLAG-HSP90α and pcDNA3-FLAG-HSP90β constructs were mutated using the Quickchange method to alter each designated amino acid. The plasmids pcDNA3-HA-HIF1α, pcDNA3-HA-HSF1 and pcDNA3-ERBB2 were constructed using a pcDNA3-TOPO directional cloning kit (Invitrogen). Other constructs were made by inserting the open reading frame of NanoLuc (NL) luciferase (Promega) upstream of the target protein in pcDNA3 by Gibson assembly according to the provided protocol (New England Biolabs). Resultant plasmids include NL-HSF1, NL-HIF1α, NL-ERBB2, NL-MET, NL-KEAP1 and NL-RHOBTB2/DBC2. For both NL-ERBB2 and NL-MET, the N-terminal extracellular domains were truncated to ensure stable expression. NL-ERBB2 was truncated to amino acid 636DLDD and NL-MET was truncated to 951GLIA. All plasmids were verified by DNA sequencing (MWG-Operon).

### Cell culture

HEK293 cells were grown in DMEM (Cellgro) with 10% fetal bovine serum (HyClone). Cells were maintained below 70% confluency in 10 cm plates. Experiments were carried with cells seeded in 96-well, 6-well, or 24-well plates (Corning). Cells were transfected using XtremeGENE 9 (Roche) for 18 hours according to the provided protocol.

### Immunoprecipitation and western blot

HEK293 cells were grown in 6-well plates to 50% confluency and transfected with XtremeGENE 9 transfection reagent according to the provided protocol. Plasmids encoding FLAG-tagged HSP90 mutant constructs were co-transfected with plasmids encoding each client protein and allowed to express for 18 hours. Cells were lysed with TGNET buffer (50 mM Tris HCl, pH 7.5, 5% Glycerol, 100 mM NaCl, 2 mM EDTA, 0.5% Triton X-100) complete with protease inhibitor cocktail and phosphatase inhibitor cocktail (Roche), then centrifuged at maximum speed for 15 minutes at 4°C. After BCA protein assay, 40 μg of protein were used as input lysate. For immunoprecipitation, 700 μg of protein from remaining lysates were added to 40 μl of anti-FLAG Beads (Sigma-Aldrich, A2220) and incubated with rotation for 2 hours at 4°C. The pull-down beads were washed 4 times with TGNET buffer. The proteins were eluted with 30 μl of 2X SDS sample buffer by boiling at 95°C for 5 min. Subsequently, samples were subjected to SDS-PAGE followed by Western blotting. The following antibodies were used in this report: FLAG (Sigma-Aldrich, A8592), HA (Rockland, #600-401-974), ERBB2 (Thermo Scientific, MS-730-P1), pERBB2 (pY1221/pY1222) (Cell Signaling, #2249), MET (Cell Signaling, #3127), pMET (Tyr1234/1235) (Cell Signaling, #3077).

### qPCR

Total RNA was isolated from heat shocked or 100 mM CoCl_2_ treated HEK293 cells using the RNeasy Mini Kit (Qiagen). One microgram of total RNA was reverse transcribed in 20 ul using the High Capacity cDNA Reverse Transcription Kit (Life Technologies), using random primers included in the kit, according to the provided protocol. The cDNA products were stored at −20°C until the PCR analysis was performed. Real-time PCR primers were designed using Primer Express software (Applied Biosystems). Each 20-uL amplification reaction contained 4 uL of diluted cDNA, 4.4 uL dH_2_O, 0.8 uL each of 5 uM forward primer and reverse primer, and 10 uL of SYBR Green PCR master mix (Life Technologies). Data were analyzed by Sequence Detection application. All targets and the control were amplified with similar PCR efficiencies. Real-time-PCR experiments were performed in triplicate. The primer sequences for real-time PCR were as follows: *DNAJ1* 5’-TGGTGCCAATGGTACCTCTTT-3’ and 5’GCCACCGAAGAACTCAGCAA-3’; *STIP1* 5’-GCAGCTACGAAACAAGCCTTCT-3’ and 5’-GACGCTGAGAGTGGTCATGATC-3’; rRNA 5’AGTCCCTGCCCTTTGTACACA-3’ and 5’- CGATCCGAGGGCCTCACTA-3’; *SLC2A1* 5’- TGGCTACAACACTGGAGTCATCA-3’ and 5’GGACCCATGTCTGGTTGTAGAACT-3’; *VEGFA* 5’TCTACCTCCACCATGCCAAGT-3’ and 5’- GATGATTCTGCCCTCCTCCTT-3’; *HSPA1A* 5’- GCCCTGATCAAGCGCAACT-3’ and 5’TTGTCGGAGTAGGTGGTGAAGA-3’

### LUMIER

LUminescence-based Mammalian IntERactome (LUMIER) assay was carried out by transfecting HEK293 cells in 96-well plates with plasmids that expressed each client protein fused to NanoLuc luciferase (Promega) along with 3XFLAG-tagged HSP90α and HSP90β. After 18 hours, cells were washed with cold PBS and lysed with 100 μl TGNET complete buffer, then centrifuged at maximum speed for 15 minutes at 4°C. The resulting lysates were transferred to an anti-FLAG antibody-coated plate (Sigma) and incubated at 4°C for 2 hours with gentle shaking. The plates were washed with TGNET. Nano-Glo reagent (Promega) was added and luciferase activity was quantified using a plate reader (Perkin-Elmer). To normalize for 3XFLAG-HSP90 levels, the plates were washed again with TGNET and anti-FLAG-HRP (Sigma-Aldrich) was added. The plates were once again incubated at 4°C, washed and assayed for HRP luminescence activity. The experimental relative interaction strength for each client protein interaction was determined by dividing the NanoLuc readout by the HRP readout value. Each sample was assayed 3 times with 3 replicates. Standard deviations are represented by error bars. A two-tailed T-test was used to determine statistical significance. All calculations were performed in Excel (Microsoft).

### Drug interaction assay

To determine the strength of binding of each HSP90 WT and mutant paralog with biotinlyated-geldanamycin (biotin-GA) and STA-7346 (biotinlyated-STA-9090), HEK293 cells were transfected with each 3XFLAG-HSP90 and allowed to express for 18 hours. Streptavidin-coated 96-well plates (Pierce) were incubated with biotinylated-geldanamycin (Sigma-Aldrich) or STA-7346 (Synta), blocked with TGNET+3% BSA and washed. Transfected cells were harvested with cold TGNET complete lysis buffer and an equal amount of fresh protein lysate was added simultaneously to a streptavidin plate and to anti-FLAG-coated plate (Sigma-Aldrich). Incubation was for 2 hours at 4°C. The plates were washed and anti-FLAG-HRP (Sigma-Aldrich) was added to each well. The plates were again incubated at 4°C for 2 hours and washed. Finally, the plates were washed and read on a plate reader using ECL to measure HRP luminescence activity (Pierce). The experimental relative interaction strength was determined by dividing the light values of the biotinlyated-drug plate by the normalizing anti-FLAG plate. Each assay was repeated 3 times with 4 replicates. Standard deviations are represented by error bars. A two-tailed T-test was used to determine statistical significance. All calculations were performed in Excel (Microsoft).
